# External validation of a nomogram for predicting tubulointerstitial lesions in IgA nephropathy: a cross-regional study in China

**DOI:** 10.1016/j.clinsp.2026.100860

**Published:** 2026-02-19

**Authors:** Xinyu Wang, Litian Pu, Lu Song, Jian Xu, Hu Meng, Meng Yang, Yan Xu, Qiao Wang, Lijuan Sun, Feifei Liu, Yaling Yu, Yaxi Zhao, Ying Shen, Qinyuan Deng

**Affiliations:** a Department of Nephrology, The First People's Hospital of Yunnan Province, Kunming University of Science and Technology Affiliated Hospital, Kunming, China; b Department of Nephrology, The First People's Hospital of Yunnan Province, School of Medicine, Kunming University of Science and Technology, Kunming, China; c Department of Integrated Traditional Chinese and Western Medicine, The First People's Hospital of Yunnan Province, Kunming University of Science and Technology Affiliated Hospital, Kunming, China; d Department of Clinical Nutrition, The Second People’s Hospital of Kunming, Kunming, China

**Keywords:** IgA nephropathy, Nomogram, External validation, Tubulointerstitial fibrosis, Altitude

## Abstract

**Background:**

A diagnostic nomogram for predicting tubulointerstitial lesions (T1/2) in IgA Nephropathy (IgAN), developed in Guangzhou (GZ) using estimated Glomerular Filtration Rate (eGFR) and Urinary Protein Excretion (UPE), demonstrated high accuracy (AUC = 0.92) but lacked external validation.

**Methods:**

The authors externally validated the nomogram in an independent cohort from Kunming (KM, n = 387; median altitude: 1,891 m), including a high-altitude subgroup (> 2,000 m, n = 155). Model performance was evaluated using Receiver Operating Characteristic (ROC) curves, calibration plots, and Decision Curve Analysis (DCA). Both creatinine-based eGFR (eGFRcr) and Creatinine-Cystatin C-based eGFR (eGFRcr-cys), each combined with UPE, were assessed.

**Results:**

The nomogram achieved nearly identical AUCs of 0.80 in the overall KM cohort for both eGFRcr-UPE (95% CI: 0.75–0.85) and eGFRcr-cys-UPE (95% CI: 0.74–0.85), demonstrating strong generalizability across eGFR formulas. In the high-altitude subgroup, performance significantly improved (AUC = 0.89; 95% CI: 0.83–0.95) for both models.

**Conclusion:**

External validation demonstrates that the nomogram combining eGFR and UPE is feasible for non-invasive prediction of T1/2 lesions, with comparable performance between eGFRcr and eGFRcr-cys. The improved accuracy observed at high altitude suggests altitude-related influences that merit further study. Prospective studies are needed to determine its prognostic value for clinical outcomes such as CKD progression and treatment response.

## Highlights

• Cross-regional validation of IgAN tubulointerstitial lesion prediction nomogram in China.
• eGFRcr and eGFRcr-cys perform equally, supporting use in low-resource settings.
• High-altitude IgAN subgroup shows improved nomogram predictive performance.

## Introduction

Immunoglobulin A Nephropathy (IgAN) accounts for approximately 12% of renal puncture biopsy cases in the United States, 25% in Europe, and up to 45% in China.^1^^,^^2^ Approximately 40% of patients with IgAN progress to End-Stage Renal Disease (ESRD).^3^ Oxford classification is the most widely applied IgAN pathology assessment System in clinical practice, and renal tubular atrophy or interstitial fibrotic lesions are not only important indicators of IgAN prognosis, but also guide clinical medication.^4^, ^5^, ^6^, ^7^, ^8^ The Validation in IgA (VALIGA) cohort showed no significant difference in the occurrence of renal endpoint events at 10-years after the combination of corticosteroids in IgAN patients with moderately active disease and significant tubulointerstitial lesions (T1/2) compared to the Renin-Angiotensin System Blockers (RASB) alone. In contrast, in IgAN patients without significant tubulointerstitial lesions (T0), RASB and corticosteroids significantly reduced the occurrence of renal endpoint events.^9^ Therefore, assessing tubulointerstitial lesions is crucial for optimizing treatment strategies in IgAN. Further research is required to refine personalized therapies based on pathological characteristics.

Renal tubulointerstitial lesions are a major tip-off for the use of corticosteroids, but renal puncture, as an invasive test, is difficult to repeat frequently. Qiongqiong Yang et al. developed a predictive model for tubulointerstitial lesions in IgAN based on estimated Glomerular Filtration Rate (eGFR) and Urinary Protein Excretion (UPE), and further constructed a diagnostic nomogram for interstitial lesions with a high predictive accuracy (AUC = 0.92, 95% CI: 0.90–0.95).^10^ However, the model distinguishes between the development set and the validation set by splitting the original cohort and does not perform external validation of the independent cohort. In external validation, AUC decreases of 0.1‒0.2 are common, up to 0.3 in extreme cases, especially when validating across regions or time.^11^ In this study, the validation cohort was based in Kunming (KM), Yunnan Province, while the development cohort of the original model was located in Guangzhou (GZ), Guangdong Province, with a straight-line distance of 1,160 km and an altitude difference of 1,880 m between the two cities. To evaluate the predictive performance and accuracy of the GZ cohort, the authors established the KM cohort by collecting data from 387 patients diagnosed with IgAN via nephropuncture and conducted external validation across regions.

## Materials and methods

### Study population

Cross-sectional data of patients diagnosed with IgAN by renal puncture from March 2023 to December 2024 were collected for this study. Systemic lupus erythematosus, purpura nephritis, hepatitis B virus-associated glomerulonephritis, and other secondary IgAN patients were excluded. Finally, 387 patients with IgAN were included in the study. This diagnostic/prognostic study was conducted and reported according to the STARD statement.

### Clinical and laboratory data

Clinical indicators of patients were collected in this study, including gender, age, height, weight and blood pressure. Laboratory data included serum creatinine, serum albumin and UPE. Body Mass Index (BMI) was calculated as weight (kg)/height (m^2^). eGFR was calculated based on the Chronic Kidney Disease Epidemiology Collaboration (CKD-EPI) formula for serum creatinine.^12^ All clinical and laboratory measurements were obtained within 1 week before renal biopsy.

### Renal pathological evaluation

Renal tissue samples for IgAN were examined by light microscopy and immunofluorescence. The Oxford Classification scoring system was used to assess and classify histological lesions, including Mesangial cell hyperplasia (M), capillary Endothelial cell hyperplasia (E), Segmental glomerulosclerosis (S), mesangial fibrosis/Tubular atrophy (T), and Cellular/fibroblastoid Crescent (C).^13^ Histopathological manifestations were independently assessed by at least two pathologists, who were blinded to all clinical and laboratory data.

### Statistical analyses

Continuous variables are expressed as mean ± standard deviation if they are normally distributed, and as median and interquartile range if they are not. Categorical variables were expressed as frequencies and percentages. Mann-Whitney or Chi-Square test was used to compare between-group differences for linked or categorical variables. Differences in Odds Ratios (ORs) between the two groups were assessed using the *Z*-test. Binary logistic regression analysis was used to assess risk factors for tubular atrophy/interstitial fibrosis. Receiver Operating Characteristic (ROC) curves and calibration curves were used to determine the predictive power and accuracy of the model. Clinically relevant operating thresholds were prespecified as 1) The Youden index-maximizing cutoff, 2) A high-sensitivity point (target sensitivity ≈90%), and 3) A high-specificity point (target specificity ≈90%). For each threshold, sensitivity, specificity, Positive Predictive Value (PPV), Negative Predictive Value (NPV), and corresponding confusion-matrix counts were calculated with 95% Confidence Intervals (95% CIs) derived from 1,000 bootstrap resamples, reported separately for the overall KM cohort and the high-altitude subgroup. R software 4.3.3 was used for statistical analysis; p < 0.05 was considered statistically significant.

## Results

### Comparison of clinical characteristics and tubulointerstitial injury between the KM and GZ cohorts

A total of 387 and 290 patients with biopsy-confirmed IgAN were included in the KM validation and GZ development cohorts, respectively (Table 1). Overall, patients in the KM cohort had a significantly higher proportion of males (42.89% vs. 32.07%, p < 0.05), higher body mass index (BMI; 23.42 vs. 22.42 kg/m², p = 0.03), and higher serum albumin levels (42.10 vs. 36.75 g/L, p < 0.05) compared to the GZ cohort. The KM cohort also had a greater proportion of patients with nephrotic-range proteinuria (UPE ≥ 3.5 g/d: 14.81% vs. 8.97%, p < 0.05), more frequent E1 (31.01% vs. 20.00%, p < 0.05) and S1 lesions (70.28% vs. 47.20%, p < 0.05), but a lower prevalence of M1 lesions (66.67% vs. 93.10%, p < 0.05). No significant inter-cohort differences were observed in age, hypertension, serum creatinine, estimated glomerular filtration rate (Both eGFRcr and eGFRcr-cys), T1/2 lesions, or C1/2 lesions (p > 0.05 for all). Although the difference was small, serum cystatin C was significantly higher in the KM cohort than in the GZ cohort (1.10 vs. 0.97 mg/L, p < 0.05).

**Table 1:** Baseline characteristics of the KM validation cohort and GZ development cohort.

Characteristic	KM validation cohort (n = 387)	GZ development cohort (n = 290)	p-value
Age (years)	34.00 (27.00, 45.00)	34 (28.00, 44.00)	0.87
Men, n (%)	166 (42.89)	93 (32.07)	<0.05
Hypertension, n (%)	131 (33.85)	98 (33.79)	0.98
BMI (kg/m^2^)	23.42 (20.81, 26.04)	22.42 (20.70, 25.13)	0.03
Serum creatinine (μmoL/L)	88.00 (67.00, 119.50)	89.00 (71.00, 125.00)	0.45
Serum cystatin c (mg/L)	1.10 (0.88, 1.52)	0.97 (0.82, 1.36)	<0.05
eGFR (KM: cr, GZ: cr-cys) (mL/min/1.73 m^2^)	82.10 (55.70, 113.60)	81.27 (50.30, 104.00)	0.12
eGFR (KM: cr-cys, GZ:cr-cys) (mL/min/1.73 m^2^)	78.72 (50.91, 104.21)	81.27 (50.30, 104.00)	0.86
Serum albumin (g/L)	42.10 (38.15, 44.85)	36.75 (32.78, 40.30)	<0.05
UPE (g/d)			<0.05
< 1	193 (50.13)	185 (63.79)	
1‒3.5	135 (35.06)	79 (27.24)	
≥ 3.5	57 (14.81)	26 (8.97)	
T1/2, n (%)	136 (35.14)	82 (28.30)	0.05
M1, n (%)	258 (66.67)	270 (93.10)	<0.05
E1, n (%)	120 (31.01)	58 (20.00)	<0.05
S1, n (%)	272 (70.28)	137 (47.20)	<0.05
C1/2, n (%)	199 (51.42)	149 (51.40)	0.99

Data are presented as median (interquartile range) or number (percentage). Comparisons were performed using the Mann-Whitney *U* test for continuous variables and the Chi-Square test for categorical variables. BMI, Body Mass Index; UPE, Urinary Protein Excretion; Egfr, estimated Glomerular Filtration Rate; eGFRcr, Creatinine-based eGFR; eGFRcr-cys, Combined Creatinine-Cystatin C-based eGFR; MEST-C scores, M (mesangial hypercellularity), E (endocapillary hypercellularity), S (segmental glomerulosclerosis), T (tubular atrophy/interstitial fibrosis), C (crescents). Serum cystatin C was missing in 45 patients (11.6%). Missing values: 2 each for UPE and BMI. p < 0.05 was considered statistically significant.

Within the KM cohort, 136 patients (35.14%) exhibited T1/2 lesions, while 251 (64.86%) were classified as T0 (Supplementary Table 1). Compared with T0 patients, those with T1/2 lesions had significantly higher prevalence of hypertension (47.79% vs. 26.29%, p < 0.05), elevated serum creatinine (122 vs. 79 μmoL/L, p < 0.05), and increased serum cystatin C levels (1.51 vs. 0.99 mg/L, p < 0.05). These patients also had markedly lower eGFR, whether estimated by creatinine (54.35 vs. 97.00 mL/min/1.73 m²) or the combined creatinine-cystatin C formula (50.21 vs. 89.79 mL/min/1.73 m²), with all differences reaching statistical significance (p < 0.05). In addition, the T1/2 group showed lower serum albumin levels (40.10 vs. 42.90 g/L, p < 0.05) and a higher prevalence of nephrotic-range proteinuria (22.39% vs. 10.76%, p < 0.05). No significant differences were noted in age, sex, or BMI between the two groups (p > 0.05).

When focusing specifically on patients with T1/2 lesions, those in the KM cohort demonstrated more favorable clinical profiles than their counterparts in the GZ cohort (Table 2). KM patients with T1/2 lesions had significantly higher eGFRcr-cys (50.21 vs. 40.21 mL/min/1.73 m^2^, p < 0.05), higher serum albumin levels (40.10 vs. 34.60 g/L, p < 0.05), and a lower prevalence of hypertension (47.79% vs. 63.41%, p < 0.05). These findings suggest that, despite comparable pathological severity, patients in the KM cohort may present with relatively preserved renal function and better nutritional status at the time of biopsy, possibly reflecting differences in clinical presentation or timing of biopsy between the two cohorts.

**Table 2:** Comparison of baseline characteristics between the KM and GZ cohorts stratified by oxford T lesions.

Variables	T0			T1/2		
	KM (n = 251)	GZ (n = 208)	p-value	KM (n = 136)	GZ (n = 82)	p-value
Age (years)	33.00 (27.00, 45.00)	33 (27.00, 42.00)	0.32	36.00 (28.00, 44.00)	37 (31.00, 46.00)	0.15
BMI (kg/m^2^)	23.70 (20.94, 26.12)	22.15 (20.53, 24.80)	<0.05	22.92 (20.42, 25.82)	23.35 (21.08, 25.80)	0.65
Serum creatinine (μmoL/L)	79.00 (62.00, 96.00)	78.00 (66.00, 96.00)	0.78	122.00 (90.00, 178.50)	173.75 (117.75, 229.75)	<0.05
Serum cystatin c (mg/L)	0.99 (0.82, 1.19)	0.88 (0.75, 1.07)	<0.05	1.51 (1.08, 2.00)	1.61 (1.28, 2.43)	0.136
eGFR (KM: cr, GZ: cr-cys) (mL/min/1.73 m^2^)	97.00 (73.00, 118.15)	95.39 (77.55, 108.14)	0.18	54.35 (38.08, 78.95)	40.21 (22.08, 61.42)	<0.05
eGFR (KM: cr-cys, GZ:cr-cys) (mL/min/1.73 m^2^)	89.79 (70.08, 110.26)	95.39 (77.55, 108.14)	0.48	50.21 (34.92, 77.34)	40.21 (22.08, 61.42)	<0.05
Serum albumin (g/L)	42.90 (39.05, 45.40)	37.50 (34.20, 40.55)	<0.05	40.10 (36.35, 43.70)	34.60 (30.28, 38.80)	<0.05
Men, n (%)	108 (43.03)	59 (28.37)	<0.05	58 (42.65)	34 (41.46)	0.88
Hypertension, n (%)	66 (26.29)	46 (22.12)	0.31	65 (47.79)	52 (63.41)	<0.05
UPE (g/d)			<0.05			0.90
< 1	154 (61.35)	164 (78.84)		39 (29.10)	21 (25.61)	
1‒3.5	70 (27.89)	38 (18.27)		65 (48.51)	41 (50.00)	
≥ 3.5	27 (10.76)	6 (2.88)		30 (22.39)	20 (24.39)	

Data are presented as median (interquartile range) or number (percentage). T0, No significant tubular atrophy/interstitial fibrosis; T1/2, Moderate-to-severe tubular atrophy/interstitial fibrosis. Comparisons between cohorts were performed using the Mann-Whitney *U* test for continuous variables and the Chi-Square test for categorical variables. BMI, Body Mass Index; UPE, Urinary Protein Excretion; eGFRcr, Creatinine-based estimated Glomerular Filtration Rate; eGFRcr-cys, Combined Creatinine-Cystatin C-based eGFR. Serum cystatin C was missing in 45 patients (11.6%). Missing values: 2 each for UPE and BMI. p < 0.05 was considered statistically significant.

### Risk factors for Oxford T lesions identified by logistic regression in the KM and GZ cohorts

Risk factors for tubular atrophy and interstitial fibrosis were assessed by multivariate logistic regression in the KM cohort. Two separate models were constructed to avoid collinearity between renal function indicators: one included eGFRcr and the other eGFRcr-cys. In both models, moderate-range proteinuria (UPE 1–3.5 g/d) and lower eGFR remained independent predictors of T-lesions (OR = 2.24 and 2.13 for UPE 1–3.5 g/d; OR = 0.97 for both eGFRcr and eGFRcr-cys; all p < 0.05). In contrast, other factors such as hypertension, serum creatinine, cystatin-C, and albumin were not statistically significant after adjustment. Notably, nephrotic-range proteinuria showed a positive association with T-lesions in the eGFRcr-cys model (OR = 1.51, p < 0.05), but not in the eGFRcr model. These findings underscore the combined importance of proteinuria and reduced kidney function in predicting tubulointerstitial injury in the KM cohort (Supplementary Table 2).

A cross-cohort comparison revealed notable differences in the strength of associations with Oxford T-lesions. In univariate analyses, both moderate-range (UPE 1–3.5 g/d) and nephrotic-range (UPE ≥ 3.5 g/d) proteinuria were more strongly associated with T1/2 lesions in the GZ cohort compared to the KM cohort (OR = 8.43 vs. 3.67 for 1–3.5 g/d, p = 0.046; OR = 26.03 vs. 4.39 for ≥ 3.5 g/d, p < 0.05). Similarly, reduced eGFR showed a stronger inverse association with tubulointerstitial damage in the GZ cohort, with lower odds ratios observed for both eGFRcr and eGFRcr-cys (0.94 vs. 0.97, p < 0.05). These inter-cohort differences were attenuated after multivariable adjustment. For moderate-range proteinuria, the between-cohort difference was no longer statistically significant in either model (Model 1: OR = 3.49 vs. 2.24, p = 0.398; Model 2: OR = 3.49 vs. 2.13, p = 0.343). The association between nephrotic-range proteinuria and T-lesions remained stronger in the GZ cohort across models (OR = 6.98 vs. 1.48 and 1.51; p = 0.088 and 0.094, respectively), though the inter-cohort difference did not reach significance. Importantly, lower eGFR remained an independent predictor of T-lesions in both cohorts in Model 1, with a statistically significant inter-cohort difference in effect size (p = 0.031), indicating a potentially stronger impact of renal dysfunction on tubulointerstitial injury in the GZ population. In Model 2, this difference approached but did not reach significance (p = 0.063), reinforcing the overall trend (Table 3).

**Table 3:** Comparison of odds ratios for variables associated with oxford T-lesions between the KM and GZ cohorts.

Variables	Univariate			Multivariate^1^			Multivariate^2^		
	KM OR (95% CI)	GZ OR (95% CI)	p-value	KM OR (95% CI)	GZ OR (95% CI)	p-value	KM OR (95% CI)	GZ OR (95% CI)	p-value
UPE (g/d)									
< 1									
1‒3.5	3.67 (2.25 ∼ 5.97)	8.43 (4.53 ∼ 16.14)	0.046	2.24 (1.22 ∼ 4.10)	3.49 (1.53 ∼ 8.05)	0.398	2.13 (1.16 ∼ 3.90)	3.49 (1.53 ∼ 8.05)	0.343
≥ 3.5	4.39 (2.34 ∼ 8.22)	26.03 (9.91 ∼ 78.17)	<0.05	1.48 (0.59 ∼ 3.72)	6.98 (1.63 ∼ 34.66)	0.088	1.51 (0.61 ∼ 3.77)	6.98 (1.63 ∼ 34.66)	0.094
eGFR (mL/min/1.73 m^2^)	0.97 (0.96 ∼ 0.97)	0.94 (0.92 ∼ 0.95)	<0.05	0.97 (0.96 ∼ 0.98)	0.95 (0.93 ∼ 0.96)	0.031	0.97 (0.95 ∼ 0.98)	0.95 (0.93 ∼ 0.96)	0.063

Odds ratios (ORs) and 95% Confidence Intervals (95% CIs) for predictors of Oxford T1/2 lesions were obtained from univariate and multivariate logistic regression models in the KM (Kunming) and GZ (Guangzhou) cohorts. Model 1 incorporated eGFRcr, and Model 2 incorporated eGFRcr-cys in the KM cohort; both models used eGFRcr-cys in the GZ cohort to avoid collinearity. All multivariate models were adjusted for hypertension, serum creatinine, serum albumin, Urinary Protein Excretion (UPE), and eGFR, with variables selected based on univariate significance (p < 0.05). p-values represent inter-cohort differences in OR magnitude, calculated using *Z*-tests. EGFRcr, Creatinine-based estimated Glomerular Filtration Rate; eGFRcr-cys, Combined Creatinine-Cystatin C-based eGFR. p < 0.05 was considered statistically significant.

In the high-altitude KM subgroup (altitude >2000 m, n = 155), stratified analysis revealed consistent trends. Among the 44 patients with T1/2 lesions (28.39%), serum creatinine (132.00 vs. 79.00 μmoL/L) and cystatin-C (1.64 vs. 1.02 mg/L) were significantly elevated, while eGFR was markedly reduced based on both creatinine (48.20 vs. 95.10 mL/min/1.73 m^2^) and creatinine-cystatin C equations (45.17 vs. 87.79 mL/min/1.73 m^2^) (all p < 0.05). Serum albumin was lower in the T1/2 group (40.40 vs. 43.10 g/L, p < 0.05), and a higher proportion had moderate-range proteinuria (56.82% vs. 29.73%, p < 0.05). Interestingly, hypertension was more prevalent in the T0 group than in the T1/2 group (74.77% vs. 52.27%, p < 0.05). No significant differences were observed in age, sex, or BMI. These findings further support the roles of reduced eGFR and moderate-range proteinuria as consistent predictors of tubulointerstitial injury, even within high-altitude settings (Table 4).

**Table 4:** Baseline characteristics of patients in the KM cohort subgroup (> 2000 m altitude), stratified by oxford T-score.

Variables	Total (n = 155)	T0 (n = 111)	T1/2 (n = 44)	p-value
Age (years)	35.00 (29.00, 45.00)	35.00 (28.00, 46.00)	35.50 (30.00, 41.00)	0.978
Men, n (%)	61 (39.35)	43 (38.74)	18 (40.91)	0.803
Hypertension, n (%)	106 (68.39)	83 (74.77)	23 (52.27)	<0.05
BMI (kg/m^2^)	23.85 (20.81, 26.44)	23.98 (20.89, 26.49)	23.44 (20.70, 25.65)	0.519
Serum creatinine (μmoL/L)	88.00 (67.00, 113.00)	79.00 (62.00, 96.50)	132.00 (102.00, 191.00)	<0.05
Serum cystatin c (mg/L)	1.12 (0.90, 1.43)	1.02 (0.82, 1.16)	1.64 (1.25, 2.02)	<0.05
eGFRcr (mL/min/1.73 m^2^)	81.80 (58.70, 108.60)	95.10 (75.30, 116.20)	48.20 (36.70, 61.93)	<0.05
eGFRcr-cys (mL/min/1.73 m^2^)	77.74 (57.11, 101.12)	87.79 (70.68, 108.98)	45.17 (33.42, 60.32)	<0.05
Serum albumin (g/L)	42.50 (39.25, 45.05)	43.10 (40.55, 45.35)	40.40 (37.15, 43.78)	<0.05
UPE (g/d)				<0.05
< 1	81 (52.26)	67 (60.36)	14 (31.82)	
1‒3.5	58 (37.42)	33 (29.73)	25 (56.82)	
≥ 3.5	16 (10.32)	11 (9.91)	5 (11.36)	

Data are presented as median (interquartile range) or number (percentage). Comparisons were performed using Mann-Whitney or Chi-Square tests. T, Tubular atrophy/interstitial fibrosis; BMI, Body Mass Index; UPE, Urinary Protein Excretion; eGFR, Estimated Glomerular Filtration Rate; eGFRcr, Creatinine-Based eGFR; eGFRcr-cys, Combined Creatinine-Cystatin C-Based eGFR. Cystatin C data were missing for 13 patients (8.4%). p < 0.05 was considered statistically significant.

### Performance of nomogram models in predicting Oxford T lesions in the KM cohort and high-altitude subgroup

**Fig. 1:**
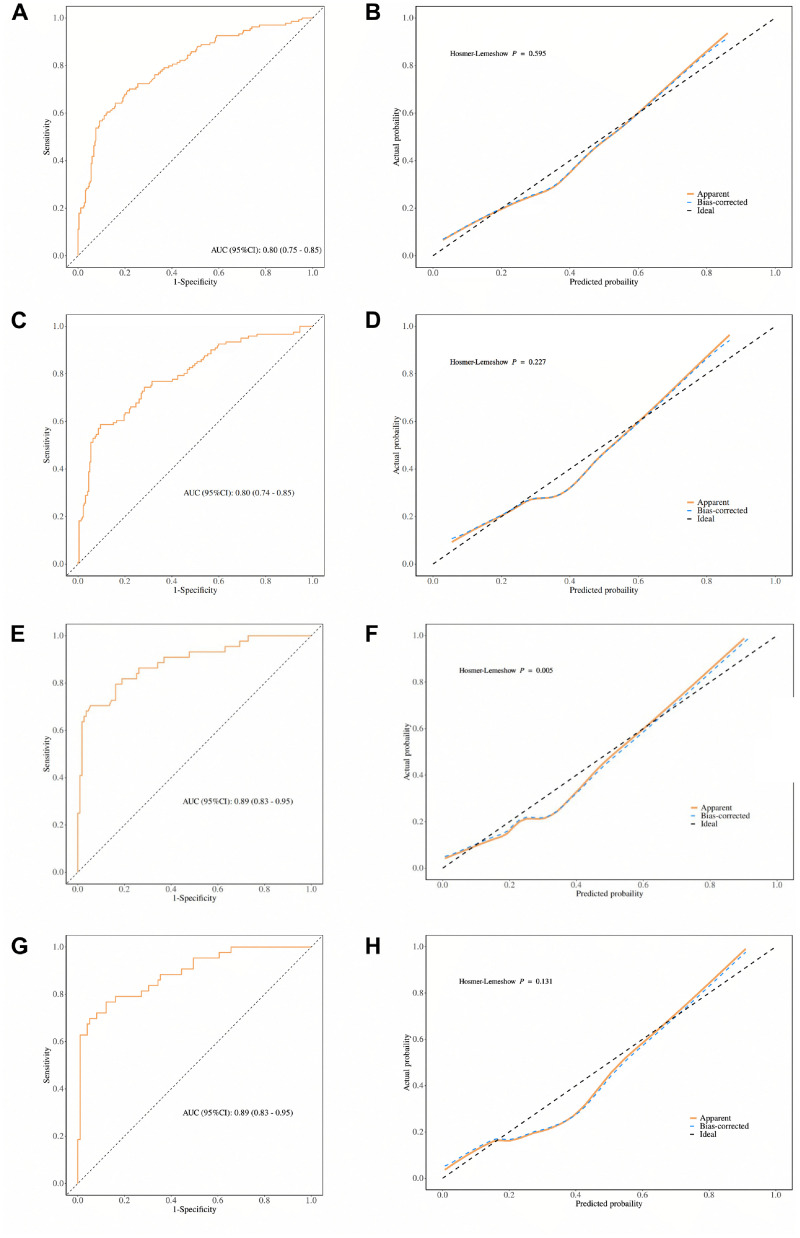
Receiver operating characteristic (ROC) and calibration curves for the nomogram based on eGFR and UPE in the KM cohort and high-altitude subgroup. (A) ROC curve of the nomogram based on eGFRcr and Urinary Protein Excretion (UPE) in the KM validation cohort. (B) Calibration curve assessing the agreement between predicted and observed probabilities using eGFRcr and UPE in the KM validation cohort. (C) ROC curve of the nomogram based on eGFRcr-cys and UPE in the KM validation cohort. (D) Calibration curve assessing the agreement between predicted and observed probabilities using eGFRcr-cys and UPE in the KM validation cohort. (E) ROC curve of the nomogram based on eGFRcr and UPE in the high-altitude KM subgroup (altitude >2000 m). (F) Calibration curve assessing the agreement between predicted and observed probabilities using eGFRcr and UPE in the high-altitude KM subgroup. (G) ROC curve of the nomogram based on eGFRcr-cys and UPE in the high-altitude KM subgroup (altitude >2000 m). (H) Calibration curve assessing the agreement between predicted and observed probabilities using eGFRcr-cys and UPE in the high-altitude KM subgroup. In all calibration plots, the dashed diagonal line represents perfect prediction. The solid orange line indicates the apparent model performance, while the blue line represents the bias-corrected curve. Calibration was evaluated using the Hosmer-Lemeshow goodness-of-fit test, with the corresponding p-values shown in each panel.

In the KM cohort, the predictive model based solely on UPE showed modest discrimination in the unadjusted analysis, with an AUC of 0.67 (95% CI: 0.61-0.72) (Supplementary Fig. 1A). Upon adding eGFR, the model’s discriminative ability improved substantially. The nomogram incorporating eGFRcr and UPE yielded an AUC of 0.80 (95% CI: 0.75–0.85), while the model using eGFRcr-cys and UPE achieved a comparable AUC of 0.80 (95% CI: 0.74–0.85) (Fig. 1A and 1C). These results remained consistent after 1,000 bootstrap resamples, with corresponding AUCs of 0.79 (95% CI: 0.74–0.84) and 0.79 (95% CI: 0.73–0.84), as shown in Supplementary Fig. 1B and 1C, respectively, confirming model robustness. Calibration plots demonstrated good agreement between predicted and observed probabilities, with Hosmer-Lemeshow test p-values of 0.595 and 0.227, respectively (Fig. 1B and 1D).

In the high-altitude subgroup (altitude > 2000 m), both nomograms demonstrated enhanced predictive performance. The models combining UPE with either eGFRcr or eGFRcr-cys each achieved an AUC of 0.89 (95% CI: 0.83–0.95), as shown in Fig. 1E and 1G. These results remained consistent after 1,000 bootstrap resamples, with identical AUCs of 0.89 (95% CI: 0.83–0.95) in Supplementary Fig. 1D and 1E, confirming model robustness. Calibration was generally accceptable, with Hosmer-Lemeshow p-values of 0.005 and 0.131, respectively (Fig. 1F and 1H). At prespecified clinical operating thresholds, consolidated diagnostic metrics, including sensitivity, specificity, PPV, NPV, and confusion-matrix counts, are provided for both models in the overall KM cohort and the high-altitude subgroup (Supplementary Table 3). These threshold-level summaries complement AUC and calibration by facilitating clinical interpretation and deployment.

**Fig. 2:**
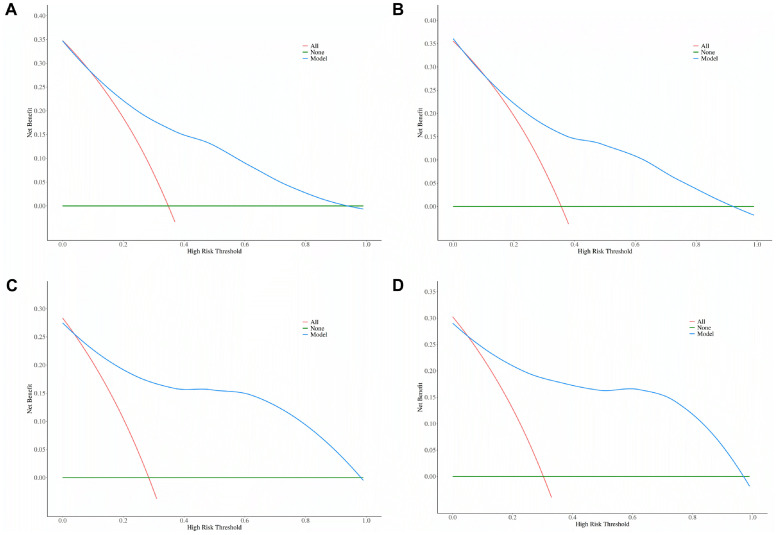
Decision curve analysis (DCA) of Nomogram Models in the KM Cohort and High-Altitude Subgroup. (A) DCA for the KM validation cohort based on eGFRcr and Urinary Protein Excretion (UPE). (B) DCA for the KM cohort using eGFRcr-cys and UPE. (C) DCA for the high-altitude subgroup of the KM cohort (altitude >2000 m) based on eGFRcr and UPE. (D) DCA for the high-altitude subgroup using eGFRcr-cys and UPE. Decision curve analysis was performed to evaluate the clinical usefulness of the prediction models by calculating the net benefit across a range of risk thresholds. The net benefit was compared between using the nomogram and alternative strategies across clinically relevant probability thresholds.

Decision curve analysis further supported the clinical utility of these models (Fig. 2). In both the KM cohort and the high-altitude subgroup, models incorporating eGFR and UPE demonstrated superior net benefit across a wide range of threshold probabilities (∼10% to 75%), compared with default strategies of assuming all or no patients have tubulointerstitial lesions. The net benefit remained consistently favorable in the high-altitude subgroup, highlighting the models’ value for individualized risk prediction of tubulointerstitial injury in IgAN.

Together, these findings highlight the added predictive value of eGFR when combined with UPE, particularly in high-altitude patients, and support the use of these nomograms for clinical risk stratification across geographically distinct IgAN populations.

## Discussion

This study presents the first external validation of a diagnostic nomogram for tubulointerstitial lesions (T1/2) in IgAN across geographically distinct Chinese cohorts, including explicit altitude stratification (median altitude of the KM cohort: 1,891 m; high-altitude subgroup: > 2,000 m). The nomogram, originally developed in the low-altitude GZ cohort using eGFRcr-cys combined with UPE (AUC = 0.92; 95% CI: 0.90–0.95), demonstrated robust predictive performance in the KM validation cohort (AUC = 0.80 for both eGFRcr and eGFRcr-cys combined with UPE; 95% CI: 0.75–0.85). The comparable AUCs across eGFR formulas support their potential interchangeability in clinical settings where cystatin-C may not be routinely available. Notably, model performance was further enhanced in the high-altitude KM subgroup (AUC = 0.89; 95% CI: 0.83–0.95), offsetting the typical 0.1–0.2 AUC decline often observed in cross-regional validations due to methodological or population heterogeneity.^11^ These findings support the nomogram’s generalizability and utility for risk stratification of tubulointerstitial injury in diverse IgAN populations.

### Key factors influencing model performance

Marked OR disparities between the KM and GZ cohorts ‒ such as the stronger association between UPE and T-lesions in GZ (OR = 8.43 vs. 3.67 for UPE 1–3.5 g/d) ‒ suggest regional differences in disease severity and response to risk factors. Although multivariable adjustment reduced these differences, residual gaps remained, particularly for nephrotic-range proteinuria and eGFR.

These differences likely reflect underlying clinical and pathological variation. The KM cohort had more males, higher BMI, and more S1 lesions, while the GZ cohort showed higher M1 scores and lower eGFR and albumin at biopsy ‒ indicating more advanced disease.^14^ Environmental and genetic influences may also contribute; proteomic studies have identified pro-inflammatory proteins associated with IgAN progression, which may vary by region.^15^ Additionally, disparities in healthcare access could delay diagnosis in some areas, amplifying the observed effect sizes.^16^ Together, these findings highlight the need to validate predictive models across diverse populations to ensure robustness and clinical relevance.

### Generalizability and practicality of creatinine-based eGFR

The authors first applied the eGFRcr formula for external validation of the nomogram, whereas the GZ development cohort used the eGFRcr-cys. Although serum creatinine is widely accessible and commonly used in clinical practice, it is influenced by non-GFR determinants such as muscle mass, age, sex, and dietary protein intake.^12^ These factors vary across regions and populations, potentially introducing bias into eGFR estimation. For example, the KM cohort had a higher mean BMI and a greater proportion of male patients than the GZ cohort, which may have elevated serum creatinine levels independent of true renal function, leading to misclassification of tubular injury severity. This concern is particularly relevant in early-stage CKD, where cystatin C, a biomarker less affected by muscle mass may provide a more accurate estimation of GFR and stronger correlation with histological damage.^17^^,^^18^ Indeed, previous studies have shown that creatinine-based eGFR tends to overestimate renal function in individuals with low muscle mass (e.g., the elderly or malnourished) and underestimate it in those with higher muscle mass or obesity.^18^, ^19^, ^20^

Nevertheless, in the present study, incorporating cystatin C into the eGFR calculation did not meaningfully improve model performance within the KM cohort. Both nomograms, based on eGFRcr with UPE and eGFRcr-cys with UPE, yielded identical AUC values of 0.80, suggesting equivalent predictive power. This finding supports the generalizability of the nomogram and indicates that creatinine-based eGFR may be sufficient for identifying tubulointerstitial lesions when combined with proteinuria. Moreover, the reliance on creatinine offers practical advantages, particularly in regions where cystatin C testing is not routinely available. Serum creatinine remains one of the most accessible and cost-effective renal biomarkers worldwide, especially in low-resource settings and primary care facilities lacking advanced laboratory infrastructure.^21^^,^^22^ The widespread use of creatinine-based equations such as CKD-EPI also ensures cross-study comparability and aligns with current clinical practice guidelines emphasizing simplicity, feasibility, and scalability.^23^, ^24^, ^25^ In such settings, cystatin C testing may not be economically feasible, further underscoring the relevance of creatinine-based tools. The structure of the present nomogram, based on creatinine and UPE, helps bridge this diagnostic gap and enables effective risk stratification even when advanced assays are unavailable. This aligns with the World Health Organization’s task-shifting strategy, which promotes the use of accessible technologies to improve healthcare delivery in underserved populations.^26^

### Enhanced nomogram performance at high altitude (>2000 m)

The enhanced performance of the nomogram at high altitude (AUC = 0.89 vs. 0.80 in the overall KM cohort) likely stems from hypoxia-driven amplification of renal injury signals and attenuation of metabolic confounders. Chronic exposure to high-altitude hypoxia has been shown to impair renal function, even in healthy individuals, resulting in reduced eGFR, increased proteinuria, and altered hemodynamics independent of comorbidities.^27^ The kidney’s high oxygen demand and blood flow make it particularly vulnerable to hypobaric hypoxia.^28^ In the present study, patients with T1/2 lesions at high altitude exhibited more pronounced declines in eGFR and a higher prevalence of moderate-range proteinuria, enhancing the discriminative power of these markers. Meanwhile, traditional confounders such as hypertension were less prevalent in this subgroup, and BMI remained comparable, allowing a more direct correlation between renal dysfunction and histopathological injury.

These findings may also reflect underlying disparities in socioeconomic status and healthcare accessibility in high-altitude regions. In Yunnan province, for instance, income and medical infrastructure are relatively limited in many mountainous and rural areas. As a result, most renal biopsies in the province are performed in tertiary centers located in Kunming, the provincial capital. This referral pattern may lead to a concentration of more severe or advanced cases in the KM cohort. Such disparities are consistent with broader evidence from Low- and Lower-Middle-Income Countries (LLMICs), where chronic kidney disease is often diagnosed late, poorly managed, and associated with higher morbidity and mortality due to limited access to early intervention and kidney replacement therapy.^29^^,^^30^ Furthermore, in these settings, systematic CKD screening is frequently infeasible due to resource constraints, making targeted case identification and effective downstream management all the more critical.^31^

Decision curve analysis further confirmed superior net benefit in the high-altitude subgroup, and bootstrap validation demonstrated excellent model stability (ΔAUC ≤0.01). Together, these findings underscore the nomogram’s clinical utility in high-altitude, resource-constrained environments, where hypoxia amplifies biomarker fidelity and socioeconomic realities necessitate practical, accessible risk assessment tools.

### Limitations and future directions

This study has several limitations. First, its cross-sectional design precludes evaluation of longitudinal outcomes such as eGFR decline, progression to ESRD, or treatment response.

Second, although both cohorts used the eGFRcr-cys formula, unmeasured factors such as regional variation in muscle mass, diet, or ethnicity may still influence biomarker levels and risk classification. Residual confounding also remains possible, since dietary composition and protein intake can modulate glomerular hemodynamics and proteinuria, while genetic background may interact with dietary exposures to alter kidney function trajectories, as shown in population-based studies.^32^ High-altitude investigations also demonstrated that both creatinine- and cystatin C–based eGFR estimates decline with increasing elevation, partly due to hematocrit changes and hypoxia-related physiology.^33^ Moreover, recent evidence indicates that creatinine- and cystatin C–based eGFR, though highly correlated, often yield discrepant CKD staging in clinical practice, reflecting the influence of unmeasured factors such as age, body composition, or inflammation.^34^

Third, important variables, including body composition, genetic or proteomic data, were not collected, limiting mechanistic interpretation, particularly regarding altitude-related effects. In addition, Socioeconomic Status (SES), a known driver of CKD progression, is not captured in the present dataset. In regions like Yunnan, where income and healthcare access vary widely, future studies should incorporate SES indicators such as household income or insurance status to better evaluate disparities in disease burden and care access. Recent biopsy data from Western China also highlight regional variation in glomerular disease patterns across age, sex, and ethnicity,^35^ further supporting the need for geographic model recalibration.

Finally, this study was limited to Chinese cohorts, and the performance of the nomogram in non–East Asian populations remains unknown. Prospective studies are warranted to validate the nomogram in multiethnic cohorts and to assess its prognostic value over time.

## Conclusion

This external validation confirms that the nomogram combining eGFR and UPE achieves moderate discriminative performance (AUC = 0.80) for predicting tubulointerstitial lesions in geographically distinct IgAN populations, supporting its feasibility as a non-invasive risk stratification tool. The comparable performance between creatinine-based and creatinine-cystatin C-based eGFR equations highlights the model’s flexibility and practical utility, particularly in resource-limited settings where cystatin-C is not routinely available. The significantly enhanced accuracy observed in high-altitude subgroups (AUC = 0.89) further suggests altitude-related amplification of renal injury signals, reinforcing the model’s clinical relevance and prompting further investigation into altitude-modulated pathophysiological mechanisms. Future longitudinal studies are warranted to determine whether the nomogram can predict clinically meaningful outcomes, such as CKD progression or treatment response.

## Declarations

ChatGPT (OpenAI) was used solely to enhance the linguistic clarity and readability of the manuscript during the writing process. No AI tools were used for data analysis, interpretation of results, or generation of scientific content. All scientific analyses and conclusions were conducted and validated by the authors.

## Availability of data and materials

The datasets generated and/or analyzed during the current study are publicly available in the Zenodo repository at: https://doi.org/10.5281/zenodo.15663338.

## Ethical approval

This study was approved by the Ethics Committee of The First People's Hospital of Yunnan Province (Approval no. KHLL2025-KY117). For all participants, written informed consent was obtained prior to inclusion.

## Funding

This research was supported by the Science and Technology Plan Project of the First People's Hospital of Yunnan Province (No. KHBS-2024-014 and KHBS-2024-012), the 10.13039/501100001809National Natural Science Foundation of China (No. 82460150), the Yunnan Fundamental Research Projects (No. 202501AU070020), and the Ten Thousand Talent Plan for Medical Expert of Yunnan Province (No. YNWR-MY-2018-019).

## Data availability statement

The datasets generated and/or analyzed during the current study are available from the corresponding author upon reasonable request.

## CRediT authorship contribution statement

**Xinyu Wang:** Conceptualization, Writing – original draft. **Litian Pu:** Formal analysis. **Lu Song:** Formal analysis. **Jian Xu:** Writing – original draft. **Hu Meng:** Data curation, Visualization. **Meng Yang:** Data curation, Visualization. **Yan Xu:** Data curation, Visualization. **Qiao Wang:** Data curation, Visualization. **Lijuan Sun:** Data curation, Visualization. **Feifei Liu:** Data curation, Visualization. **Yaling Yu:** Data curation, Visualization. **Yaxi Zhao:** Formal analysis, Data curation, Visualization. **Ying Shen:** Conceptualization. **Qinyuan Deng:** Formal analysis, Writing – original draft.

## Declaration of competing interest

The authors declare no conflicts of interest.

## Appendix Supplementary materials

Image, application 1

## Footnotes

Supplementary material associated with this article can be found, in the online version, at doi:10.1016/j.clinsp.2026.100860.
