# Supplementary material for: External validation of a nomogram for predicting tubulointerstitial lesions in IgA nephropathy: a cross-regional study in China
Source: Clinics (Sao Paulo). 2026 Feb 19;81:100860. doi: 10.1016/j.clinsp.2026.100860 (PMC12933437; doi:10.1016/j.clinsp.2026.100860)

**CLINICS-D-25-00534_ Supplementary Material**

**Supplementary Table 1** Baseline characteristics of the KM cohort stratified by oxford T-lesions in IgA nephropathy patients.

| **Variables** | **Total (n = 387)** | **T0 (n = 251)** | **T1/2 (n = 136)** | **p-value** |
| --- | --- | --- | --- | --- |
| Age (years) | 34.00 (27.00, 45.00) | 33.00 (27.00, 45.00) | 36.00 (28.00, 44.00) | 0.267 |
| Men, n (%) | 166 (42.89) | 108 (43.03) | 58 (42.65) | 0.942 |
| Hypertension, n (%) | 131 (33.85) | 66 (26.29) | 65 (47.79) | <0.05 |
| BMI (kg/m^2^) | 23.42 (20.81, 26.04) | 23.70 (20.94, 26.12) | 22.92 (20.42, 25.82) | 0.222 |
| Serum creatinine (μmoL/L) | 88.00 (67.00, 119.50) | 79.00 (62.00, 96.00) | 122.00 (90.00, 178.50) | <0.05 |
| Serum cystatin c (mg/L) | 1.10 (0.88, 1.52) | 0.99 (0.82, 1.19) | 1.51 (1.08, 2.00) | <0.05 |
| eGFRcr (mL/min/1.73 m^2^) | 82.10 (55.70, 113.60) | 97.00 (73.00, 118.15) | 54.35 (38.08, 78.95) | <0.05 |
| eGFRcr-cys (mL/min/1.73 m^2^) | 78.72 (50.91, 104.21) | 89.79 (70.08, 110.26) | 50.21 (34.92, 77.34) | <0.05 |
| Serum albumin (g/L) | 42.10 (38.15, 44.85) | 42.90 (39.05, 45.40) | 40.10 (36.35, 43.70) | <0.05 |
| UPE (g/d) |  |  |  | <0.05 |
| < 1 | 193 (50.13) | 154 (61.35) | 39 (29.10) |  |
| 1‒3.5 | 135 (35.06) | 70 (27.89) | 65 (48.51) |  |
| ≥ 3.5 | 57 (14.81) | 27 (10.76) | 30 (22.39) |  |

Data are presented as median (interquartile range) or number (percentage). T0, No significant tubular atrophy/interstitial fibrosis; T1/2, Moderate-to-severe tubular atrophy/interstitial fibrosis. Comparisons were performed using the Mann-Whitney *U* test for continuous variables and the Chi-Square test for categorical variables. BMI, Body Mass Index; UPE, Urinary Protein Excretion; eGFRcr, Creatinine-Based estimated Glomerular Filtration Rate; eGFRcr-cys, combined Creatinine-Cystatin C-based eGFR. Serum cystatin C was missing in 45-patients (11.6%); Missing values, 2 each for UPE and BMI. p-value < 0.05 was considered statistically significant.

**Supplementary Table 2** Multivariate logistic regression analysis of variables associated with oxford T-lesions in the KM cohort.

| **Variables** | **With eGFRcr** | | **With eGFRcr-cys** | |
| --- | --- | --- | --- | --- |
|  | **OR (95%CI)** | **p-value** | **OR (95%CI)** | **p-value** |
| Men, n (%) | 0.78 (0.44 ~ 1.40) | 0.407 | 0.76 (0.43 ~ 1.35) | 0.344 |
| Hypertension, n (%) | 1.38 (0.76 ~ 2.49) | 0.293 | 1.35 (0.75 ~ 2.46) | 0.318 |
| UPE (g/d) |  |  |  |  |
| < 1 | 1.00 (Reference) |  | 1.00 (Reference) |  |
| 1‒3.5 | 2.24 (1.22 ~ 4.10) | <0.05 | 2.13 (1.16 ~ 3.90) | <0.05 |
| ≥ 3.5 | 1.48 (0.59 ~ 3.72) | 0.406 | 1.51 (0.61 ~ 3.77) | <0.05 |
| Age (years) | 0.98 (0.96 ~ 1.01) | 0.133 | 0.98 (0.96 ~ 1.01) | 0.169 |
| Serum creatinine (mg/dL) | 1.00 (1.00 ~ 1.01) | 0.480 | 1.00 (1.00 ~ 1.01) | 0.290 |
| Serum cystatin c (mg/L) | 1.01 (0.78 ~ 1.30) | 0.963 | 0.80 (0.58 ~ 1.11) | 0.180 |
| Serum albumin (g/L) | 0.97 (0.92 ~ 1.03) | 0.281 | 0.98 (0.93 ~ 1.04) | 0.491 |
| eGFRcr (mL/min/1.73m^2^) | 0.97 (0.96 ~ 0.98) | <0.05 | NA | NA |
| eGFRcr-cys (mL/min/1.73m^2^) | NA | NA | 0.97 (0.95 ~ 0.98) | <0.05 |

Odds ratios (ORs) and 95% Confidence Intervals (95% CIs) were derived from multivariate logistic regression models evaluating the association between clinical variables and Oxford T1/2 lesions in the KM cohort. Two separate models were constructed to avoid collinearity: one including eGFRcr (creatinine-based estimated Glomerular Filtration Rate), and the other including eGFRcr-cys (combined Creatinine-Cystatin C-based eGFR). Both models were adjusted for hypertension, serum creatinine, serum albumin, Urinary Protein Excretion (UPE), and additional covariates with p < 0.05 in univariate analyses. p-values < 0.05 were considered statistically significant.

**Supplementary Table 3** Diagnostic performance of eGFR-UPE nomograms in the overall KM cohort and the high-altitude subgroup.

| **Cohort & Model** | **AUC (95% CI)** | **Accuracy (95% CI)** | **Sensitivity (95% CI)** | **Specificity (95% CI)** | **PPV (95% CI)** | **NPV (95% CI)** | **Cut off** |
| --- | --- | --- | --- | --- | --- | --- | --- |
| Overall KM |  |  |  |  |  |  |  |
| eGFRcr + UPE | 0.80 (0.75‒0.85) | 0.76 (0.71‒0.80) | 0.79 (0.74‒0.84) | 0.69 (0.62‒0.77) | 0.83 (0.78‒0.88) | 0.64 (0.56‒0.71) | 0.39 |
| eGFRcr-cys + UPE | 0.80 (0.74‒0.85) | 0.79 (0.74‒0.83) | 0.90 (0.87‒0.94) | 0.59 (0.50‒0.67) | 0.80 (0.75‒0.85) | 0.77 (0.69‒0.86) | 0.50 |
| altitude >2000 m |  |  |  |  |  |  |  |
| eGFRcr + UPE | 0.89 (0.83‒0.95) | 0.88 (0.82‒0.92) | 0.95 (0.90‒0.99) | 0.70 (0.57‒0.84) | 0.89 (0.83‒0.95) | 0.84 (0.72‒0.96) | 0.49 |
| eGFRcr-cys + UPE | 0.89 (0.83‒0.95) | 0.87 (0.81‒0.92) | 0.95 (0.91‒0.99) | 0.70 (0.56‒0.83) | 0.88 (0.82‒0.94) | 0.86 (0.74‒0.97) | 0.55 |

Diagnostic accuracy metrics of the nomogram models combining eGFR and Urinary Protein Excretion (UPE). Results are presented for both creatinine-based (eGFRcr + UPE) and creatinine-cystatin C-based (eGFRcr-cys + UPE) equations in the overall KM cohort and the high-altitude subgroup (>2000 m). Values are shown with 95% Confidence Intervals (95% CI). Cutoffs correspond to the optimal thresholds derived from the Youden index within each cohort. Abbreviations: AUC, Area Under the Curve; PPV, Positive Predictive Value; NPV, Negative Predictive Value; eGFRcr, estimated Glomerular Filtration Rate based on creatinine; eGFRcr-cys, estimated Glomerular Filtration Rate based on combined Creatinine and Cystatin C; UPE, Urinary Protein Excretion; KM, Kunming cohort.

**Supplementary Figure 1** Receiver Operating Characteristic (ROC) Curves Based on UPE and eGFR in the KM Cohort and High-Altitude Subgroup (Bootstrapped with 1000 Resamples). (A) ROC curve of the model based on Urinary Protein Excretion (UPE) alone in the KM validation cohort (without bootstrap resampling). (B) ROC curve of the nomogram based on eGFRcr and UPE in the KM cohort, validated using 1000 bootstrap resamples. (C) ROC curve of the nomogram based on eGFRcr-cys and UPE in the KM cohort, validated using 1000 bootstrap resamples. (D) ROC curve of the nomogram based on eGFRcr and UPE in the high-altitude KM subgroup (altitude > 2000 m), validated using 1000 bootstrap resamples. (E) ROC curve of the nomogram based on eGFRcr-cys and UPE in the high-altitude KM subgroup (altitude >2000 m), validated using 1000 bootstrap resamples.


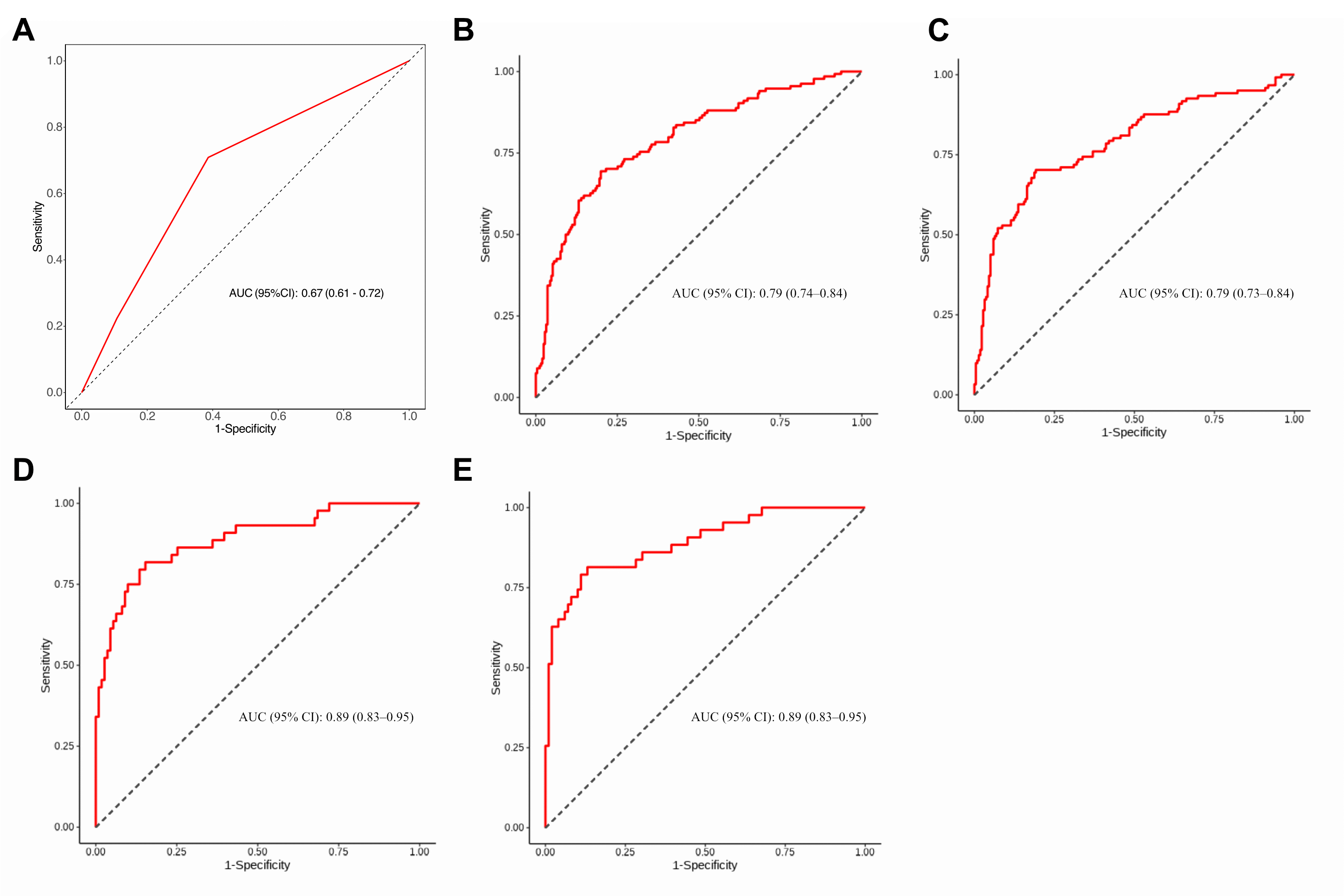

Supplement: Supplementary file 1 [file mmc1.docx]
